# Broadband Low-Cost Normal Magnetic Field Probe for PCB Near-Field Measurement

**DOI:** 10.3390/s25133874

**Published:** 2025-06-21

**Authors:** Ruichen Luo, Zheng He, Lixiao Wang

**Affiliations:** 1 School of Information Engineering, Xiamen Ocean Vocational College, Xiamen 361005, China; luoruichen@stu.xmu.edu.cn; 2 Fujian Engineering Research Center for Electronic Design Automation (EDA), Institute of Electromagnetics and Acoustics, Xiamen University, Xiamen 361005, China; hezheng@stu.xmu.edu.cn; 3 College of Information Science & Technology, Eastern Institute of Technology, Ningbo 315200, China

**Keywords:** broadband measurement, electromagnetic radiation characterization, magnetic field detection, microstrip line calibration, near-field probe, RF circuit diagnostics

## Abstract

This paper presents a broadband near-field probe designed for measuring the normal magnetic field ($H_{z}$) in radio frequency (RF) circuits operating within a frequency range of 2–8 GHz. The proposed probe uses a cost-effective 4-layer printed circuit board (PCB) structure made with an FR-4 substrate. The probe primarily consists of an $H_{z}$ detection unit, a broadband microstrip balun, and a coaxial-like output. The broadband balun facilitates the conversion from differential to single-ended signals, thereby enhancing the probe’s common-mode rejection capability. This design ensures that the probe achieves both cost efficiency and high broadband measurement performance. Additionally, this work investigates the feasibility of employing microstrip lines as calibration standards for the $H_{z}$ probe. The probe’s structural parameters and magnetic field response were initially determined through simulations, and the calibration factor was subsequently verified by calibration experiments. In practical measurements, the field distributions above a microstrip line and a low-noise amplifier (LNA) were captured. The measured field distribution of the microstrip line was compared with simulation results to verify the probe’s performance. Meanwhile, the measured field distribution of the LNA was utilized to identify the radiating components within the amplifier.

## 1. Introduction

In complex electromagnetic environments, electronic products should maintain stable operation free from interference while ensuring that their near-field and far-field emissions remain non-disruptive to other devices. With continuous advances in chip integration and component miniaturization, along with rising operational frequencies in modern devices, the physical dimensions of circuit boards, packages, and integrated circuits are now approaching the wavelengths of their fundamental frequencies or harmonics. This dimensional-electromagnetic interaction transforms these components into unintended radiating elements, significantly impacting electromagnetic compatibility (EMC). As a result, electromagnetic interference (EMI) issues have become more severe [1]. As a result, near-field scanning-based EMI diagnostic systems have emerged as crucial tools to ensure successful certification and minimize compliance adjustment efforts [2,3]. Within these near-field scanning systems, electric and magnetic field probes serve as critical components, as they directly determine overall measurement accuracy and diagnostic efficiency.

**Figure 1:**
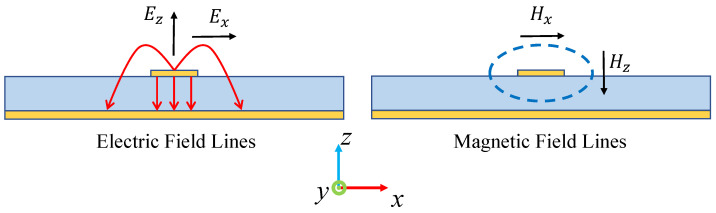
Electromagnetic field components generated by a microstrip line.

Typically, such probes are classified according to their detection mechanism, electric or magnetic. As shown in Figure 1, the electromagnetic fields generated by transmission line structures (exemplified by microstrip lines) decompose into orthogonal components in Cartesian coordinates. Accordingly, measurement probes are divided into different types based on the specific field characteristics being measured ($E_{x}$, $E_{y}$, $E_{z}$, $H_{x}$, $H_{y}$, and $H_{z}$).

Substantial research efforts have been devoted to electric field probes [4,5,6] and magnetic probes [7,8,9,10,11] for measuring specific electromagnetic field components. Broadband probes demonstrate stable performance across wide frequency ranges, which is particularly crucial for identifying unknown radiation sources in EMI diagnosis. These designs primarily accommodate various devices under test (DUT). When the radiation frequency is uncertain, these broadband probes identify the actual radiating band, thereby facilitating subsequent analysis and improvements. In addition to these broadband solutions, narrow-band probes [12,13,14] offer high sensitivity within specific frequency bands through the use of resonant structures. Although sacrificing bandwidth, these probes enable sensitive detection of weak signals within known frequency ranges, which proves particularly valuable for targeted EMI suppression in pre-characterized systems. To address the time constraints of robotic scanning systems, particularly when mapping large-area circuits or complex multilayer boards, multi-component probes [15,16,17,18] have been developed. These advanced designs simultaneously capture multiple field components during a single scanning pass, effectively reducing measurement time.

The near-field distribution data acquired through near-field probes can be directly utilized to analyze and localize radiation positions and intensities or employed for radiation source reconstruction to derive equivalent radiation sources. These equivalent sources enable the prediction of radiation patterns at arbitrary spatial positions and coupling assessment while significantly reducing model complexity [19,20]. Various reconstruction methodologies have been investigated, with the most prevalent approaches employing ideal point source (electric and magnetic dipoles) [21,22] or electric/magnetic current source-based surface integral equation [23,24]. The reconstruction process constitutes an inverse problem typically requiring matrix inversion, which represents a field-to-source transformation. Optimal reconstruction theoretically requires complete six-field-component measurements to ensure field information completeness and minimize solution ambiguity. This necessitates comprehensive field measurements using diverse probe configurations. While substantial research exists on electric field probes (measuring $E_{x}$, $E_{y}$, $E_{z}$) and magnetic field probes (measuring $H_{x}$, $H_{y}$), $H_{z}$ component magnetic probes remain understudied.

**Figure 2:**
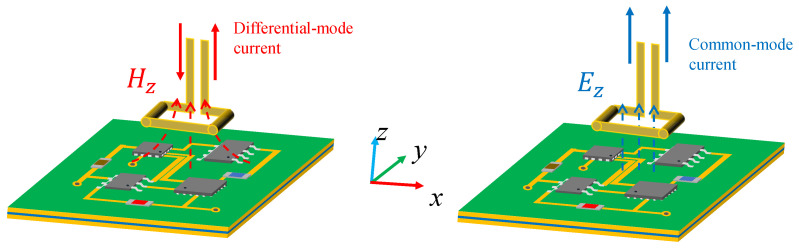
Differential and common mode currents induced by $H_{z}$ and $E_{z}$ fields.

For a probe specifically designed to measure a particular electromagnetic field component (e.g., the $H_{z}$ component), orthogonal field components (such as $E_{z}$) will introduce interference noise that compromises measurement accuracy. As shown in Figure 2, the front-end metallic loops of the $H_{z}$ probes are positioned in regions exposed to both $H_{z}$ and $E_{z}$ fields, respectively. The $H_{z}$ field induces sensing currents of equal amplitude and opposite phase in the metallic loops, forming an effective differential-mode signal. Conversely, the interfering $E_{z}$ field generates common-mode currents with identical amplitude and phase. When these components coexist, the superposition of common-mode and differential-mode signals in the transmission channel degrades signal integrity. Therefore, a balance-to-unbalance converter (balun) is necessary to suppress common-mode interference effectively. By leveraging its common-mode rejection capability, the balun effectively suppresses $E_{z}$-induced common-mode currents. This ensures reliable extraction of $H_{z}$ differential-mode signals and significantly improves the measurement signal-to-noise ratio (SNR).

In this paper, we develop a broadband near-field probe aimed at measuring the normal component of the magnetic field ($H_{z}$) in radio-frequency (RF) circuits, which cover frequencies from 2 to 8 GHz. The main contributions of this work are summarized as follows:

- An integrated near-field probe design that incorporates a microstrip-slotline balun is presented, providing effective common-mode noise suppression and enabling accurate capture of $H_{z}$ fields from 2 GHz to 8 GHz without reliance on expensive high-frequency substrates.
- The probe employs a cost-effective FR-4 substrate instead of expensive high-frequency materials for fabrication. Though it introduces a trade-off in high-frequency performance, this approach significantly reduces manufacturing costs and remains compatible with standard multilayer printed circuit board (PCB) processes.

The paper is organized as follows: Section 2 details the probe design, including structural layout and balun implementation. Section 3 presents the calibration methodology, highlighting how the calibration factor is derived from microstrip-line-based standards. In Section 4, experimental results are presented for both a microstrip line and a low-noise amplifier (LNA) circuit, demonstrating the probe’s broad applicability and effectiveness. Finally, Section 5 concludes the paper and discusses potential directions for future work.

## 2. Design of the Probe

**Figure 3:**
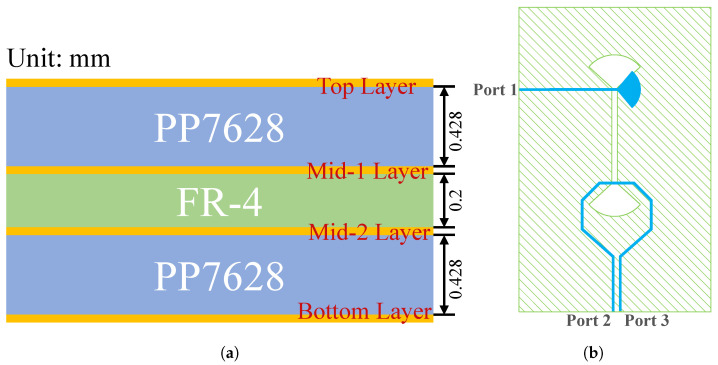
(**a**) Stack-up of the utilized four-layer FR-4 PCB. (**b**) Schematic of the microstrip-slotline-microstrip balun transition structure.

The probe consists of three key components: a front-end metallic magnetic loop, a central balun transition, and a terminal output transmission line. The entire probe is manufactured using a stacked PCB structure shown in Figure 3a. The core substrate employs FR-4 material with a relative permittivity of 4.20. Meanwhile, PP7628 prepreg sheets (with an approximate relative permittivity of 4.18) are used as bonding layers above and below the core. This material configuration ensures stable electromagnetic performance while maintaining structural integrity through the multilayer stacking process. The prepreg layers not only provide interlayer insulation but also contribute to controlled impedance characteristics of the transmission lines across different layers. These layers collectively ensure both mechanical robustness and consistent electromagnetic characteristics. The designed probe was first tested for performance using simulation software. After confirming its performance, the actual probe was fabricated and subjected to laboratory testing.

### 2.1. Structural Layout and Balun Implementation

The top and bottom layers employ 1 oz copper thickness for their metallic sections, whereas the mid-1 and mid-2 layers utilize 0.5 oz copper. Within the mid-1 and mid-2 layers, three essential elements are integrated: (1) a broadband balun; (2) a front-end metallic magnetic loop; (3) the necessary transmission lines that serve as transitions.

**Figure 4:**
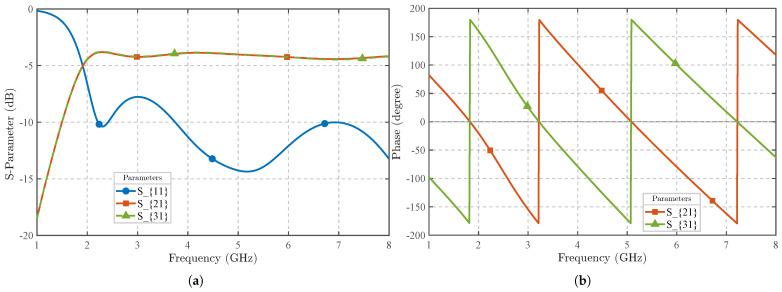
(**a**) *S*-parameters of the balun transmission line across the 2–8 GHz frequency range. (**b**) Phase response of the balun transmission line in the same frequency range.

The broadband balun is crucial for converting the front loop’s differential signal into a single-ended output across a wide frequency range [25,26]. This process extends to the output, creating a microstrip-slotline interface [27,28,29]. As shown in Figure 3b, we integrate the balun into the probe using a microstrip-slotline-microstrip transition, which achieves a 180° phase delay by reversing the electric field direction [30,31]. The microstrips at both ends of the transition structure are located on the same copper layer, which is the blue layer in the design, while the slotline is positioned on a different copper layer, the green layer. It is important to note that the slotline layer serves as the ground plane for the microstrip layer. Figure 4 demonstrates the simulated broadband performance: across the 2–8 GHz range, both $|S_{21}|$ and $|S_{31}|$ remain near −3 dB, and the phase difference between the two outputs remains at 180°, confirming the balun’s broadband performance and its suitability for the specified frequency range.

Strategically positioned to capture the magnetic field, the front-end metallic magnetic loop generates two induced currents that are equal in magnitude but opposite in phase. These currents feed into the input terminals of the balun, resulting in out-of-phase signals that propagate along the microstrip lines in opposite directions. This phase difference is crucial for converting the differential signal into a single-ended output.

**Figure 5:**
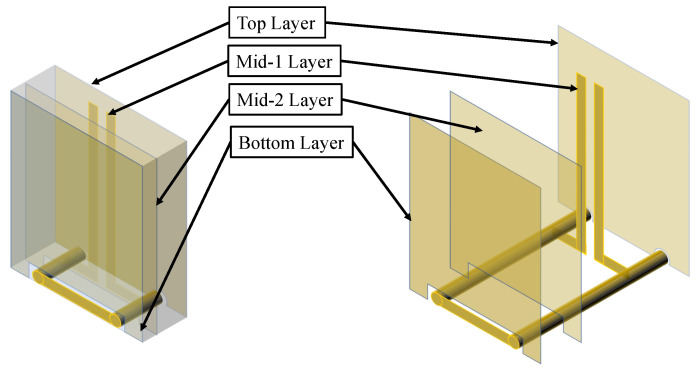
3D perspective and exploded view of the magnetic front loop.

Figure 5 illustrates the 3D perspective of the probe’s front loop structure. As the core component of the probe, the magnetic induction metal loop comprises two vertical vias interconnected with transmission lines. The torus-shaped loop exhibits a normal direction aligned with that of the measured plane, enabling effective detection of the magnetic field’s normal component.

**Figure 6:**
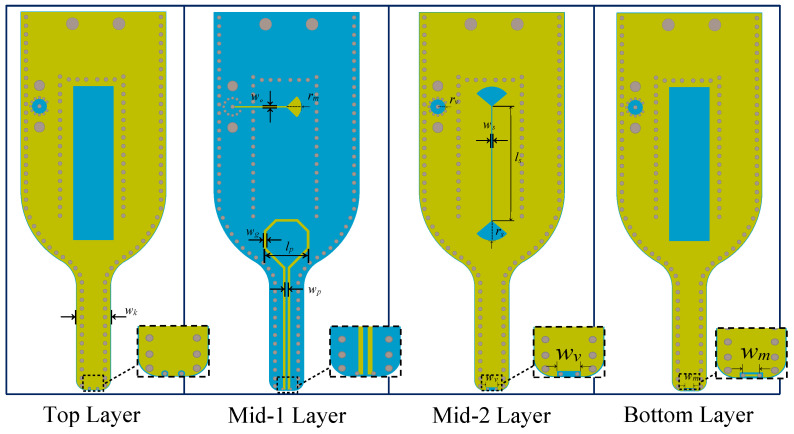
Copper layout for each layer in the 4-layer FR-4 PCB structure, highlighting the key components and physical dimensions.

Figure 6 showing each layer in a plane-by-plane schematic. In this figure, the yellow regions denote the metallic components of the probe, while the blue areas indicate dielectric substrates. The design was optimized for both bandwidth and impedance characteristics through systematic parametric sweeps, focusing on key physical dimensions such as the gap, stub, and loop diameter, which are labeled in Figure 6. In addition, Table 1 lists the final set of optimized parameter values.

During the measurement process, magnetic field information is first captured by the bottom magnetic loop and transmitted upward through differential lines to the microstrip-slotline transition structure. In this conversion stage, differential-mode signals are effectively preserved while common-mode noise undergoes significant suppression. The processed signals then propagate along the slotline until reaching a slotline-to-microstrip transition structure, which facilitates signal transfer to the output microstrip line.

The output terminal incorporates a vertical signal via surrounded by multiple shielding ground vias, both specifically designed to interface with a through-hole sub-miniature-A (SMA) connector. The coaxial-like structure formed by the signal-ground via configuration ensures impedance continuity between the stripline and the SMA connector, effectively reducing signal leakage. Above design consideration enhances measurement accuracy by maintaining signal integrity from the probe tip to the external measurement instrumentation.

### 2.2. Fabrication Considerations on FR-4 Substrate

The probe design in this study utilizes FR-4 material as the dielectric substrate instead of Rogers high-frequency series materials, primarily driven by cost control considerations. Taking Rogers RO4003C as an example, Table 2 systematically compares their key parameters: Although RO4003C demonstrates superior performance in dielectric loss (Df as low as 0.0027) and high-frequency stability (±0.05 dielectric constant tolerance), its unit cost is 5–8 times higher than FR-4. By adopting FR-4 material and appropriately relaxing transmission line performance indicators within acceptable limits, this project achieved an approximate 80% reduction in substrate cost.

**Figure 7:**
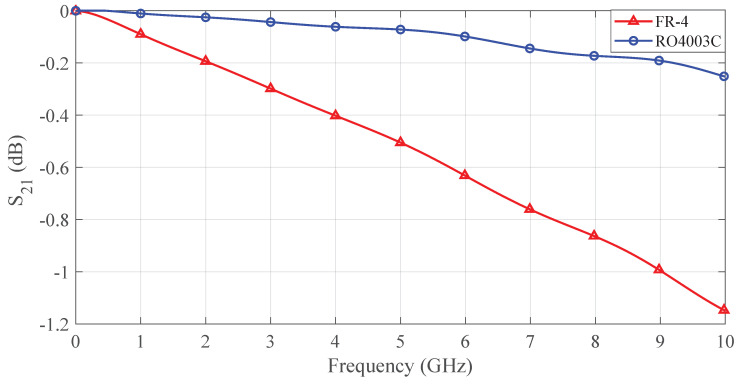
Comparison of insertion loss ($S_{21}$) by using different dielectrics.

To validate the feasibility of material substitution, this research conducted a comparative analysis of insertion loss based on a microstrip line model. Under strictly maintained line length (40 mm) and port impedance (50 Ω), the loss characteristics shown in Figure 7 were obtained through full-wave electromagnetic simulation. Simulation results demonstrate that within the 0–9 GHz operational bandwidth, both materials maintain insertion loss better than −1 dB (FR-4: −0.95 dB @9 GHz, RO4003C: −0.21 dB @9 GHz), proving the theoretical viability of FR-4 substitution in this frequency range. Considering substrate fabrication tolerances (5% dielectric constant deviation) and connector soldering losses, practical testing conservatively limited the probe’s operational bandwidth to 8 GHz, thus ensuring system robustness.

## 3. Probe Calibration

### 3.1. Derivation of the Calibration Formulation

The probe’s output is captured by instruments—most commonly a vector network analyzer (VNA)—and reported in terms of *S*-parameters, in particular the transmission coefficient $S_{21}$. However, $S_{21}$ alone does not directly represent the true strength of the radiated field. To bridge this gap, the probe must be calibrated: by comparing its $S_{21}$ readings against known field intensities, one derives a calibration factor CF that converts measured transmission coefficients into actual field strength [32].

For magnetic field probes, calibration typically employs the standardized microstrip line recommended in IEC 61967-6 [33]. This method leverages the microstrip’s analytically solvable radiation characteristics to directly correlate probe outputs with reference field values, thereby determining the CF. With advancements in simulation technology, the analytical field calculation process can now be replaced by electromagnetic simulation modeling. This approach facilitates the design and optimization of calibration fixtures, particularly for structures where analytical field solutions are unavailable.

In this work, we maintain the microstrip line as the calibration kit for the proposed probe. The calibration factor is defined as follows:

(1) $$\mathrm{CF}=\frac{|H_{z}|}{|V_{out}|}=\frac{|H_{z}|}{|S_{21}\left|\right|V_{1}|}\;\left(S/m\right)$$

(2) $$|V_{1}|=\sqrt{2\cdot P\cdot 50}$$

where *P* denotes the power at the VNA port and 50 (Ω) represents the port impedance. The port voltage $|V_{1}|$ in the simulation and measurement needs to be consistent.

This frequency-dependent calibration converts instrument readings into absolute field values across the operational bandwidth. The calibration procedure accounts for both probe sensitivity variations and system transmission losses, ensuring measurement traceability to established electromagnetic field standards.

The calibration process is as follows:

1. Obtain the reference magnetic field magnitude $|H_{z}|$ over the calibration kit by the electromagnetic simulator.
2. Assemble the measurement system.
3. Measure the $|S_{21}|$ parameter under controlled excitation of VNA, which directly correlates with the probe’s output voltage magnitude $|V_{out}|$.
4. Compute the calibration factor using the Equation (1), establishing the field-to-signal conversion relationship.

### 3.2. Simulated Model of the Probe and Microstrip Cal. Kit with Field Distribution Analysis

According to IEC 61967-6 guidelines [33], a microstrip line was chosen as the calibration kit for near-field probe calibration due to its stable and well-defined field distribution. The microstrip line features a trace width of 1 mm, a substrate thickness of 0.5 mm, and is fabricated on FR-4 with a relative permittivity of 4.20. In particular, the microstrip line supports a quasi-TEM wave propagating along the *y*-axis, resulting in minimal field variation in this direction [34,35]. This characteristic simplifies probe positioning and ensures clearly observable field peaks along the *x*-direction. Additionally, microstrip lines are cost-effective and easy to fabricate, making them practical and widely adopted calibration fixtures.

**Figure 8:**
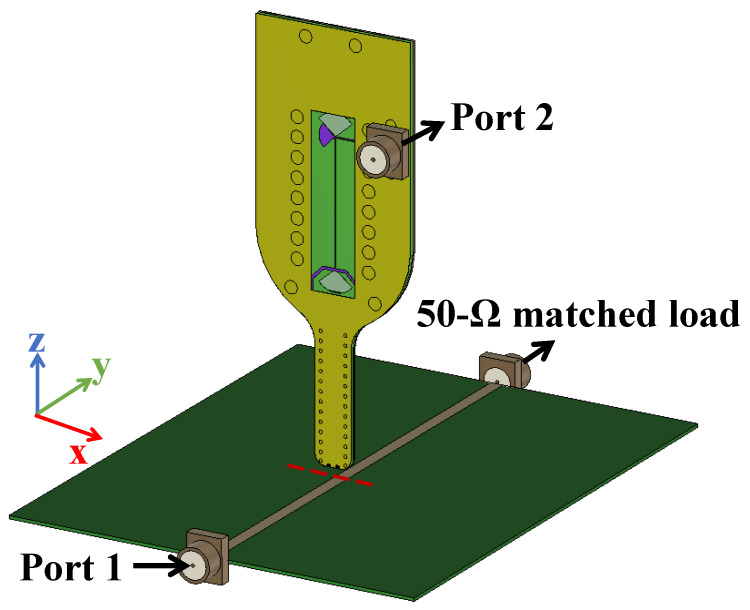
Three-dimensional electromagnetic simulation model of the microstrip line and probe, featuring all three ports.

As illustrated in Figure 8, the microstrip line was modeled in a 3D FEM-based electromagnetic solver, with all ports configured as wave ports. One end of the line is driven by a 50 Ω source, while the other is terminated in a 50 Ω load to replicate realistic feeding conditions. A probe, held 1 mm above the substrate, scans along the red dashed path parallel to the *x*-axis (the red dashed line) in 0.1 mm increments to capture high-resolution cross-sectional field distributions.

**Figure 9:**
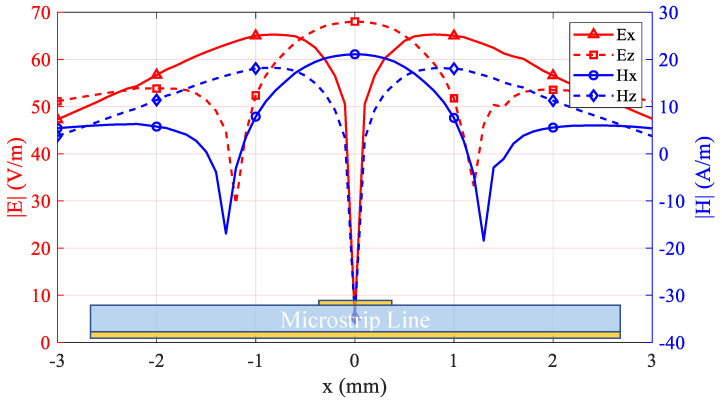
Normalized field distributions above the microstrip line at a height of 1 mm.

Figure 9 presents the simulated electric and magnetic field distributions along the *x*-axis, illustrating symmetrical patterns about the center conductor. In order to better interpret the field distribution of the microstrip line, we need to refer to Figure 1 for an explanation. In the magnetic field line model diagram of the microstrip line shown on the right side of Figure 1, the magnetic field is a closed curve surrounding the central conductor. Therefore, by observing the intensity of each component of the magnetic field (mainly $H_{x}$ and $H_{z}$) at this time, it can be seen that in the *x*-direction, the $H_{x}$ field at the center of the conductor is the strongest, and it generally shows a downward trend as it extends to both sides. For $H_{z}$, there is almost no component in the *z*-direction at the center of the conductor, so this is the zero point of $H_{z}$. When extending to both sides, $H_{z}$ gradually increases until it reaches the peak and then continues to decrease. The electric field can also be analyzed using this method.

Notably, $E_{x}$ and $H_{z}$ share peak and valley positions, as do $H_{x}$ and $E_{z}$. This correlation indicates that, in the absence of proper shielding or a balun, unintended field components (specifically $H_{x}$ and $E_{z}$ in this study) can couple into the probe and degrade measurement accuracy. In accordance with standard calibration protocols, the probe is typically placed at field peak locations to maximize the signal-to-noise ratio and enhance calibration precision.

**Figure 10:**
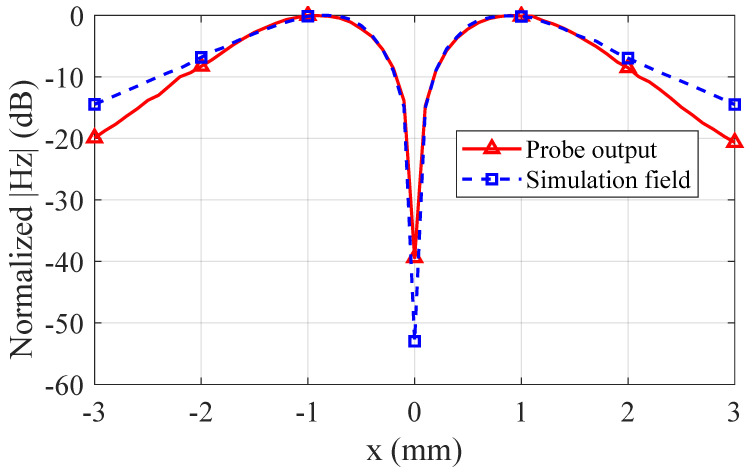
Comparison of normalized magnetic field measurements between the proposed probe and reference simulation.

Figure 10 illustrates the normalized field results for the microstrip line as obtained from the custom-designed probe and from an ideal simulation. The curves demonstrate good correlation near the microstrip centerline, validating the measurement accuracy in high-field regions. However, a gradual discrepancy appears farther from the centerline, where the probe’s output slightly underestimates the true $H_{z}$ distribution. This discrepancy arises because residual coupling from non-target field components persists despite the balun and shielding structures. The reference simulation represents an idealized scenario free from practical measurement constraints.

Although improving the probe’s peripheral sensitivity remains a valuable research direction, this work focuses on ensuring accurate measurements in high-intensity field regions—an essential requirement for reliable probe calibration. The demonstrated performance in central areas thus provides reliable calibration coefficients for practical applications, especially considering that most calibration procedures and EMC tests center on peak-field measurements.

### 3.3. Practical System Implementation and Measured Calibration Factors

**Figure 11:**
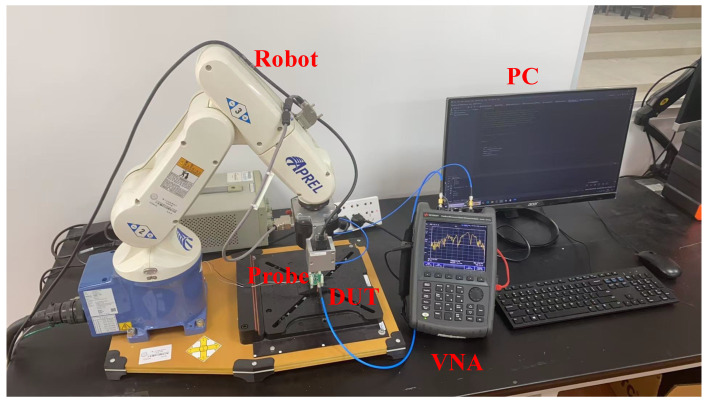
Setup of our near-field scanning system in this work.

The near-field scanning system used in this work covers the 2–8 GHz range and incorporates a mechanical scanning arm capable of wide-ranging and precise positional adjustments for accurately capturing magnetic field variations. As illustrated in Figure 11, a VNA (N9928A, Keysight Technologies, Santa Rosa, CA, USA) serves as both the excitation and receiving instrument, with Port 1 connected to the DUT and Port 2 connected to the fabricated probe for $S_{21}$ measurements.

**Figure 12:**
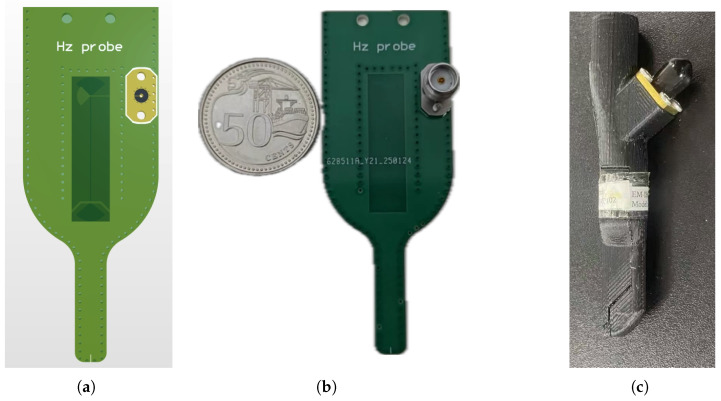
(**a**) Simulation model. (**b**) Fabricated probe. (**c**) APREL $H_{z}$ probe.

Figure 12a illustrates the simulation model of the proposed probe, and Figure 12b shows the fabricated probe alongside a 50-cent Singapore coin for size reference, where a straight-plug SMA connector is used to interface the probe with the coaxial cable. Figure 12c shows a commercially available $H_{z}$ probe—model ALS-Hz-2-6 GHz from APREL Industries, Ottawa, ON, Canada—which is specified for operation from 9 kHz to 6 GHz.

To characterize the operational bandwidth, the proposed probe is initially placed 1 mm above the microstrip line at the peak magnetic field location, and its *S*-parameter $S_{21}$ is measured.

**Figure 13:**
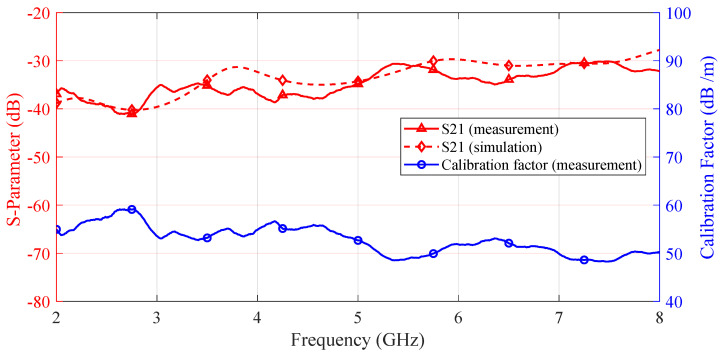
Measured *S*-parameters compared with simulated results across the 2–8 GHz range, with the derived calibration factor for probe performance.

Figure 13 presents both the measured $S_{21}$ and the corresponding simulated $S_{21}$ over the 2–8 GHz range, illustrating the discrepancy between empirical data and theoretical predictions. From these measured $S_{21}$ data, a calibration factor is derived (using Equation (1)) and is plotted in the same figure. This calibration curve serves as a quantitative reference for evaluating the probe’s measurement accuracy throughout its operational frequency range.

## 4. Measurement Results

The calibration results were presented in the previous section. Based on these findings, a series of practical measurements have been conducted to validate the performance of the probe.

### 4.1. Measurement of $H_{z}$ Field over a Microstrip Line

**Figure 14:**
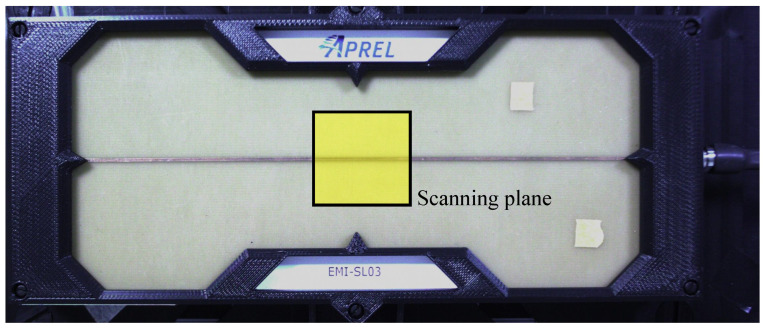
Measurement setup for the microstrip line.

We conducted radiated-field scans over the area above the microstrip line shown in Figure 14. This line is identical to the calibration microstrip described in Section 3.2 and, in this context, serves as the device under test (DUT). One end of the line is connected to Port 1 of the VNA via a coaxial cable, while the other end is terminated with a 50 Ω matched load. The probe is connected to Port 2 during the measurement.

The scanning plane, indicated in the yellow area in Figure 14, has dimensions of 24.8 mm × 11.8 mm and is sampled with a step resolution of 0.2 mm. This yields a total of 7500 scanning points (125 × 60). Each complete scan took around an hour, which included not only the VNA’s frequency sweep but also the time required for probe movement, host PC I/O operations (and associated waiting times), and data processing on the host computer. During the scanning process, the probe tip was maintained at a height of 1 mm above the microstrip surface. As anticipated by theory, the maximum $H_{z}$ field was observed near the microstrip’s centerline, providing a reliable reference for validating the probe’s performance.

Simultaneously, we conducted electromagnetic simulations of the microstrip to serve as a comparison. Owing to the simplicity of the geometry and the capability of our workstation—equipped with an Intel^®^ Core™ i7 2.90 GHz processor (Intel Corporation, Santa Clara, CA, USA) and 24 GB of RAM—a full frequency-sweep simulation completes in only 11 s, after which a brief post-processing step produces the field-distribution plots.

**Figure 15:**
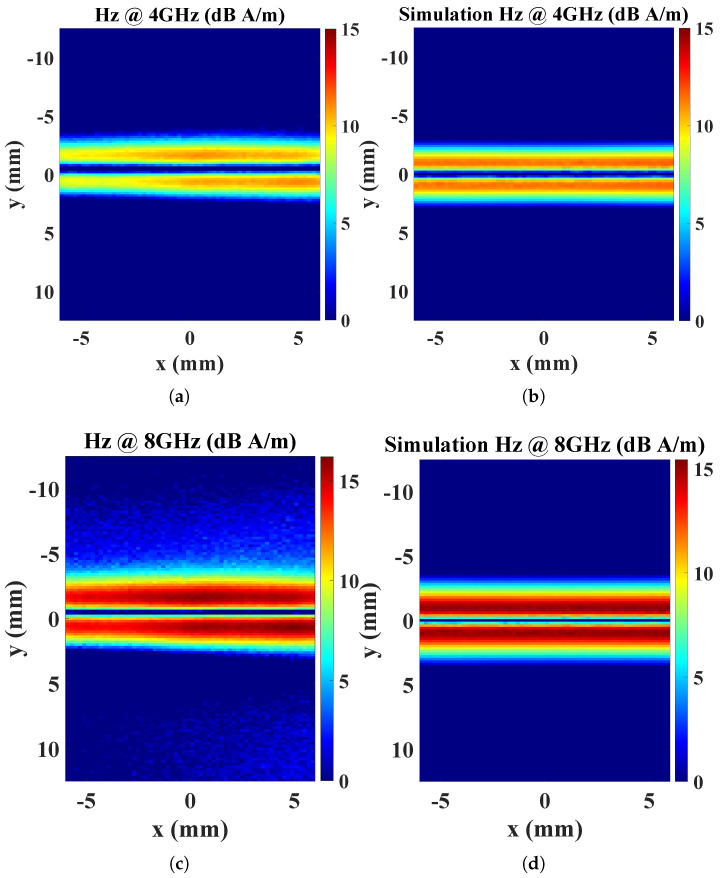
Magnetic field $H_{z}$ distributions measured by the proposed probe: (**a**) measurement results at 4 GHz; (**b**) simulation results at 4 GHz; (**c**) measurement results at 8 GHz; (**d**) simulation results at 8 GHz.

As depicted in Figure 15, both the measured and simulated $H_{z}$ field distributions at various operating frequencies reveal distinct zero and peak field positions that align well with theoretical models of microstrip line magnetic fields. Nevertheless, despite minor distortions being observed in the measured data, the overall field distributions and radiation intensities remain consistent with simulation results. These distortions may be attributed to a slight impedance mismatch between the microstrip line and SMA connectors. The consistent alignment between measured and simulated data validates the reliability of the proposed probe design and demonstrates its broadband operational capability across the tested frequency range.

### 4.2. A Comparative Analysis of the Performance Between the Proposed Probe and the APREL Commercial Probe

To quantitatively evaluate the common-mode interference suppression performance, comparative measurements were conducted on microstrip line fields using both the proposed probe and the APREL commercial probe. As theoretically predicted in the preceding analysis (Figure 9), the cross-sectional center of the microstrip line (x = 0 mm) corresponds to the minimum $H_{z}$ field intensity while simultaneously exhibiting maximum $E_{z}$ field magnitude. Elevated probe output at this critical position directly indicates compromised common-mode rejection capability.

**Figure 16:**
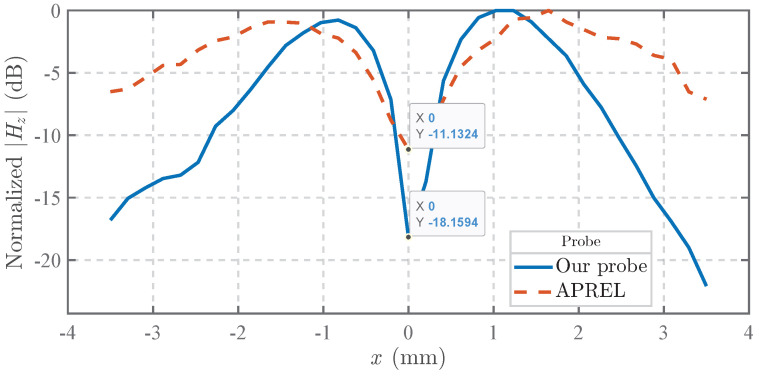
Comparison of $H_{z}$ measurements at 2 GHz for the proposed probe and the APREL commercial probe.

As shown in Figure 16, at an operating frequency of 2 GHz, the proposed probe demonstrates a significant suppression advantage with its output amplitude measuring nearly 7 dB lower than that of the APREL probe at the microstrip’s cross-sectional center. This quantifiable reduction validates the enhanced common-mode noise rejection characteristics engineered into our probe design.

**Figure 17:**
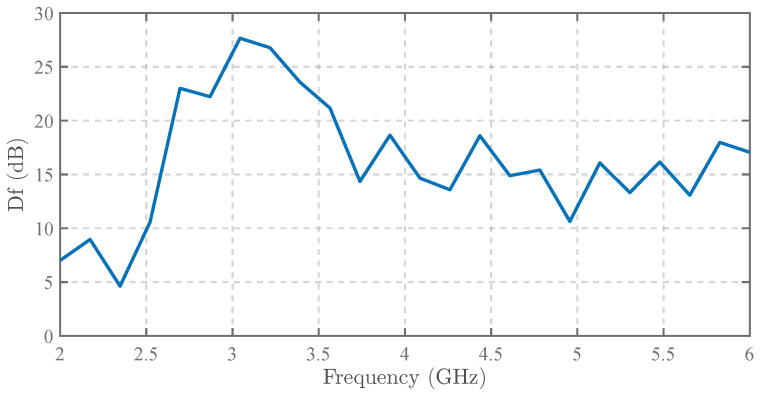
The measured common-mode suppression improvement curve (compared with the APREL probe).

For clearer visualization, following a similar approach to Figure 16, we calculated the dB-scaled difference (denoted as $D_{f}$) between the normalized outputs of both probes at x = 0 mm across frequencies, where $D_{f}$ is defined as the APREL probe’s output minus that of our designed probe. The results within 2–6 GHz are presented in Figure 17. The graph demonstrates that our probe exhibits superior common-mode interference suppression effectiveness compared to the APREL probe across the entire frequency range.

### 4.3. Measurement of $H_{z}$ Field over a Low Noise Amplifier

Previous measurements on simple microstrip line structures have established the reliability of the proposed near-field probe. However, those tests represented an oversimplified scenario. In this study, we extend our validation by investigating a functional LNA circuit, focusing on its operational magnetic field characteristics. The inductors in the driver circuit of the LNA, while essential for impedance matching and DC biasing, can inadvertently become radiating elements at RF frequencies due to both current flow and parasitic interwinding capacitances. Such effects become particularly pronounced when the inductors’ self-resonant frequencies approach the LNA’s operational band, potentially causing undesired EMI. Due to the alignment of the winding axes of the two inductive components with the normal direction of the circuit, the magnetic field predominantly concentrates in the perpendicular direction. This concentration of the magnetic field makes the use of $H_{z}$ measurements both more efficient and straightforward, with a higher signal-to-noise ratio. Hence, we specifically measure the magnetic fields around these inductors to assess both amplifier circuit performance and potential EMI risks.

**Figure 18:**
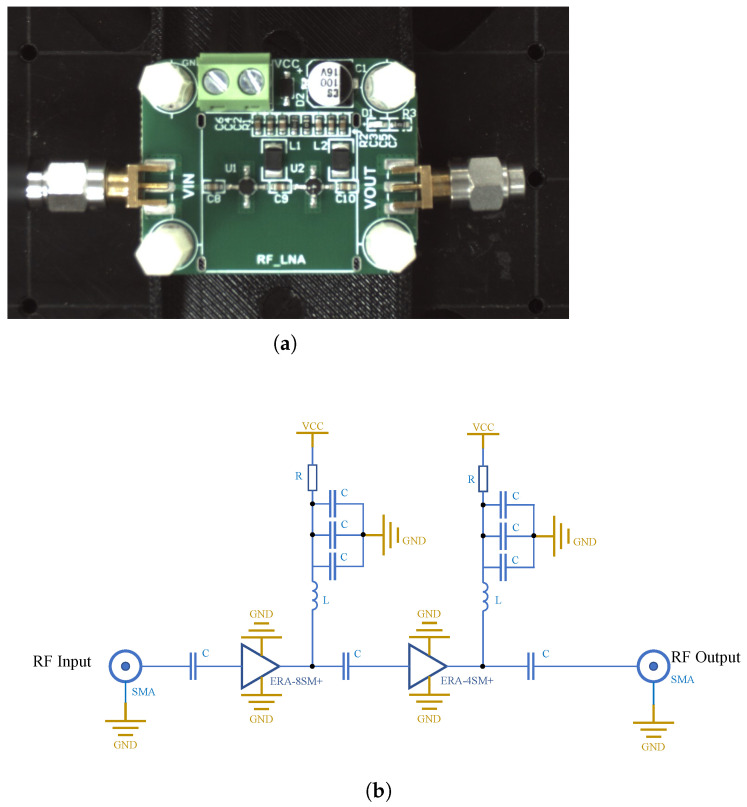
(**a**) Photograph of the LNA used in the measurement. (**b**) Schematic diagram of the LNA circuit.

Figure 18a shows the physical LNA prototype and the region scanned in our experiment, where the circuit is powered by a +5 V supply and consists of two amplifier chips (ERA-8SM+, ERA-4SM+) alongside the driver components (inductors, resistors, and capacitors). The equivalent circuit diagram is shown in Figure 18b.

To conduct magnetic near-field measurements, we placed the prototype such that one port is connected to Port 1 of a VNA, while the opposite port is terminated with a 50 Ω. The probe is attached to Port 2 of the VNA and scans an area of 28.2 mm × 12.3 mm in 0.3 mm steps, yielding a total of 3990 measurement points. The probe tip remains at a fixed 1 mm distance above the circuit surface during all scans.

**Figure 19:**
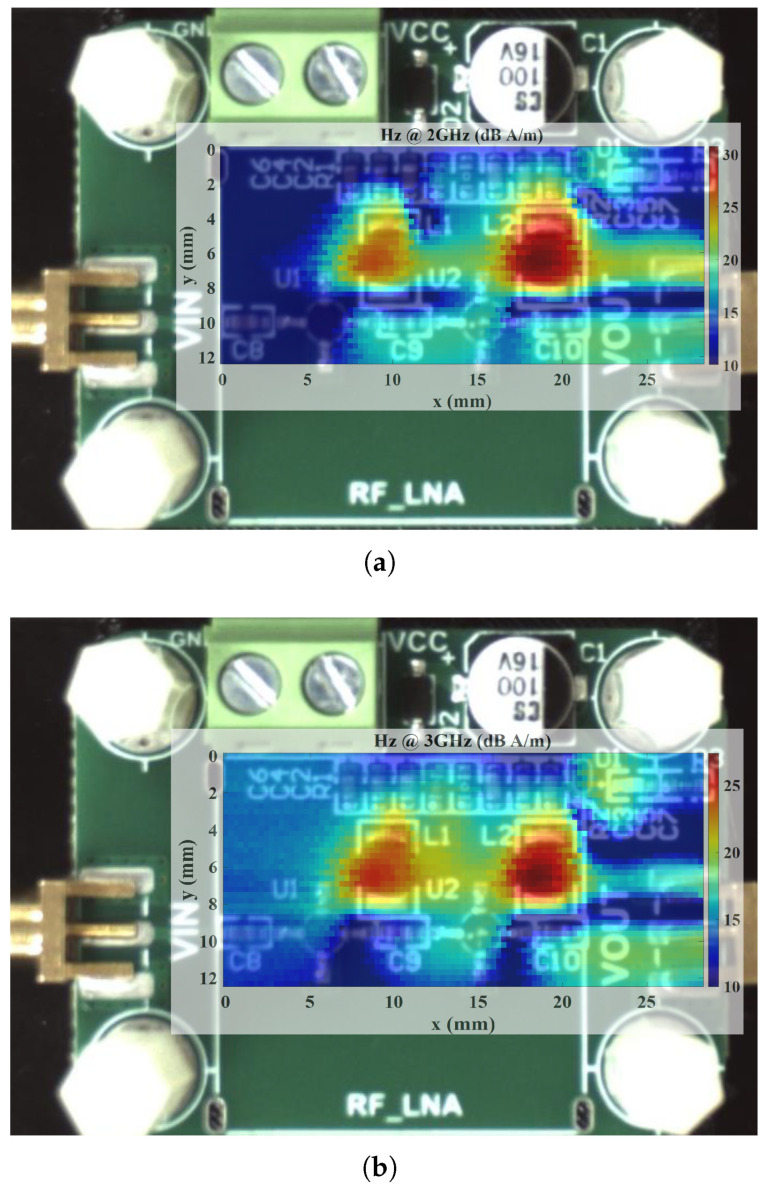
$H_{z}$ distributions above the LNA at (**a**) 2 GHz and (**b**) 3 GHz.

During the testing of the LNA, we measured the $H_{z}$ field distributions at two frequency points: 2 GHz and 3 GHz (Figure 19). The rationale behind choosing these frequencies is twofold. First, the LNA is designed to operate up to 2 GHz; consequently, the measurement at 2 GHz lies within this designated operating band and exhibits a relatively higher peak magnetic field. This observation indicates proper amplifier functionality at its intended frequency range. In contrast, the measurement at 3 GHz, beyond the LNA’s design range, shows a notably lower peak value, consistent with the expected performance degradation in non-operational frequencies. Although the proposed probe can operate across 2–8 GHz, this comparison of in-band versus out-of-band performance highlights the LNA’s behavior under both normal and off-design conditions.

Notably, the results indicate strong magnetic field radiation emanating from the two inductors in the circuit, aligning with the LNA’s schematic. The measured findings further reveal that the radiation intensity near the output port exceeds that near the input port by approximately 15 dB, reflecting the anticipated signal amplification characteristics of the amplifier stage. These observations confirm the probe’s effectiveness in detecting component-level magnetic emissions in practical RF systems.

Furthermore, these findings highlight the probe’s potential for EMI/EMC optimization [36]. Specifically, the probe’s high spatial resolution enables rapid localization of undesired radiated “hotspots” in complex RF systems, such as high-speed PCB designs, allowing engineers to quickly isolate problematic components or trace segments (e.g., inductors, vias, or coupling regions). Guided by these measurements, targeted solutions—such as refining ground loops, integrating additional shielding, or adjusting component placement—can be implemented and verified in subsequent scanning, thereby expediting the overall EMI debugging process and reducing design iteration cycles.

## 5. Conclusions

This work presents a broadband near-field probe for measuring the normal magnetic field ($H_{z}$) in RF circuits from 2 to 8 GHz. The probe integrates a microstrip-slotline balun to achieve differential-to-single-ended conversion, effectively reducing common-mode interference, thus realizing cost effectiveness in a four-layer FR-4 PCB. A microstrip line approach is employed for calibration, yielding accurate and repeatable results through a straightforward calibration factor.

Experimental evaluations on both a microstrip line and an LNA confirm the probe’s broadband stability and ability to identify localized magnetic hotspots. In particular, the measurements closely match simulations for the microstrip line, while the LNA tests reveal distinct radiation differences between input and output. These findings underscore the probe’s suitability for diagnosing EMI sources and guiding EMI/EMC improvements in practical RF systems.

By balancing cost-effectiveness with performance, this probe offers a reliable solution for near-field scanning, circuit debugging, and electromagnetic compliance assessments. However, the low-frequency response of the proposed probe is somewhat limited by the balun’s performance, which reduces its sensitivity below approximately 2 GHz. Future work may focus on improving the balun structure to enhance low-band performance, extending the design to higher frequencies, and incorporating advanced shielding techniques to reduce undesired coupling.

## Figures and Tables

**Table 1:** List of optimized design parameters for the proposed probe.

Parameters	$w_{k}$	$w_{o}$	$r_{m}$	$w_{g}$	$l_{p}$	$w_{p}$
Value (mm)	5.8	0.2	2.5	0.4	7.8	0.4
Parameters	$r_{v}$	$w_{s}$	$l_{s}$	$r_{s}$	$w_{v}$	$w_{m}$
Value (mm)	1.3	0.2	19.8	3.5	2	1.4

**Table 2:** Comparison of key material properties between FR-4 and RO4003C substrates.

Material	FR-4	RO4003C
Unit Cost	Low (1/8–1/5 of RO4003C cost)	High (5–8 times the FR-4 cost)
HF Performance	Moderate (High loss, ±15% $\epsilon_{r}$ variation)	Superior (Low loss, ±0.05 $\epsilon_{r}$ tolerance)
Application Scope	Low-frequency, cost-sensitive designs	High-performance systems, microwave
Fabrication Complexity	Standard PCB processes	Requires controlled lamination

## Data Availability

Data are contained within the article.
